# Determinants of loneliness and depressive symptomatology among LGBTQ older adults living with cognitive impairment

**DOI:** 10.1002/alz.091912

**Published:** 2025-01-09

**Authors:** Hyun‐Jun Kim, Karen I Fredriksen‐Goldsen, Hailey H. Jung, Chaejeong Lee

**Affiliations:** ^1^ University of Washington, Seattle, WA USA

## Abstract

**Background:**

Loneliness and depression among older adults are linked to a higher likelihood of chronic diseases, deterioration of physical function, and compromised quality of life. LGBTQ older adults are known to experience social isolation and mental distress at higher rates than their heterosexual counterparts, and those with cognitive impairment may be particularly susceptible to loneliness and depression. However, there is limited knowledge regarding the risk and protective factors for loneliness and depression among LGBTQ older adults living with cognitive impairment. This study fills this critical research gap and provides insights to improve the well‐being of this historically marginalized and underserved population.

**Method:**

A sub‐sample (N = 160) of the Aging with Pride: National Health, Aging, and Sexuality/Gender Study (NHAS), the first longitudinal study of LGBTQ adults aged 50 and older in the U.S., was used. Those who were identified as experiencing cognitive impairment based on their Montreal Cognitive Assessment (MOCA) scores were included in this study. The MOCA scores ranged from 14 to 25 with a mean score of 22.1 (SD = 2.7). Mixed models were tested to identify factors predicting the overall levels and slopes of loneliness and depressive symptomatology over 6 years (2014 – 2020).

**Result:**

The mean age was 68 (range: 50 – 97) with 69% men, 44% people of color, 55% with income below 200% federal poverty guideline, and 22% with high school or less education. Half reported feeling lonely, and about 30% had clinical‐level depressive symptoms. After controlling for background characteristics, loneliness and depressive symptomatology were positively associated with everyday discrimination, sub‐optimal sleep, and limited social resources. Loneliness was also positively associated with being single and limited outdoor activity, and depressive symptomatology with identity stigma and malnutrition. Loneliness increased over time if being single.

**Conclusion:**

Findings identify modifiable factors that may provide protection against loneliness and depressive symptomatology. Individuals living with cognitive impairment and no care partner had loneliness increase over time, which is of particular concern. This study provides evidence‐based information for the development of tailored interventions, addressing LGBTQ‐specific needs as well as enhancing independence in daily living and overall quality of life.

